# Phytochemical Diversity Comparison in Leaves and Roots of Wild and Micropropagated Latvian Sea Holly (*Eryngium maritimum* L.)

**DOI:** 10.3390/molecules28093924

**Published:** 2023-05-06

**Authors:** Ilva Nakurte, Marta Berga, Ieva Mežaka

**Affiliations:** Institute for Environmental Solutions, “Lidlauks”, Priekuli Parish, LV-4126 Cesis, Latvia

**Keywords:** plant tissue culture, phytochemical screening, secondary metabolites, essential oils, triterpenoid saponins, rosmarinic acid, amino acids, headspace gas chromatography

## Abstract

The goal of the current study was to compare the chemical composition of the roots, shoots, and leaves of wild-growing *Eryngium maritimum* L., and of in vitro and in field-cultivated plants in Latvia. The essential oil yield obtained by hydrodistillation ranged from 0.14% to 0.54%, while analysis of the chemical composition using GC-MS revealed a total of 44 different volatiles, with differences in the types and amounts of volatiles between the leaves and roots. Using 96-well plate techniques, the concentration of total phenolic compounds, saponins, and sugars in the aqueous ethanolic extracts of *E. maritimum* were assessed, along with their capacity to scavenge stable DPPH radicals. Extracts from roots had a lower concentration of total phenolic compounds compared to those from the leaves of wild grown and cultivated plants but did not differ from in vitro shoots. Root, leaf, and shoot samples of the same genotype from different growth conditions had approximately the same concentration of total saponins, while total sugar concentrations were higher in the roots. The growth conditions had a significant effect on the concentration of total phenolic compounds and antiradical activity, with differences that were significant observed between plant aboveground and belowground parts. Analysis using UHPLC-ESI-q-TOF-MS revealed 63 compounds, with amino acids and hydroxycinnamic acid derivatives (such as chlorogenic and rosmarinic acid) being the major compound groups that significantly differed between plant growth conditions. We also demonstrated that rapid screening of volatile compounds in in vitro plants using headspace gas chromatography mass spectrometry analyses can predict the formation of marker compounds in the same mericlones grown in field conditions. These findings provide valuable insights into the chemical composition of *E. maritimum* and its potential for use in various applications.

## 1. Introduction

*E. maritimum*, also known as sea holly, is a plant species that grows in coastal areas of Europe and northern Africa, covering the Black Sea, the Mediterranean, the Atlantic coasts of North Africa and Europe, and the coasts of the North Sea and Baltic Sea [1]. Latvia and Estonia are the northern borders of the distribution range of sea holly [2]. It is considered an endangered species in Europe, including Latvia, due to the destruction of its habitat and excessive harvesting from the wild. It is forbidden to collect herbs for commercial purposes in the wild because they are identified as needing protection in the Red Data Book of the Baltic Region [3] because of issues such as habitat degradation, excessive harvesting, and disruption of coastal ecosystems. In addition to Latvia, several countries such as the UK, France, Italy [4], and Poland [5] also consider sea holly to be an endangered or vulnerable species. Efforts are being made to conserve and protect sea holly populations in Europe, including through habitat restoration and protection measures as well as through regulations on the harvesting and trade of the plant. For those reasons, biotechnological techniques such as in vitro cultures can be used for the conservation and propagation of these endangered plant species [6,7,8,9,10]. Alternatively, efforts can be made to cultivate sea holly under controlled conditions in order to obtain its phytochemicals without contributing to the depletion of wild populations.

Used as ornamentals or vegetables, *E. maritimum* also has a long history of use in traditional medicine for a variety of purposes, including as a digestive aid, diuretic, and anti-inflammatory agent [11,12]. Additionally, the plant has been used to treat conditions such as fever and bronchitis [13]. Potential anticancer properties, such as the capability of *E. maritimum* to reduce the toxicity associated with chemotherapeutic agents such as cisplatin [14], as well as enzymatic inhibitory properties [15], have also been described. Due to its antimicrobial, antioxidant, hepatoprotective, and anti-inflammatory properties [11,12,13,16], sea holly appears to be an excellent candidate for use as a cosmetic additive [17] and is already used as an extract that is added to formulations such as lotions, creams, and serums [18,19]. Leaves and roots of the plant are edible, but its commercial use is generally limited to small-scale, local consumption [20]. However, some of these species have been consumed in some traditional dishes in certain regions [21,22]. The commercial exploitation of the species for the food industry is hindered by the slow adaptation of plants to changes in costal ecosystems [20], as well as the endangered status of northern populations [5].

Due to *E. maritimum’s* endangered status, it is important to ensure that any harvesting of sea holly is carried out sustainably and with appropriate permits and regulations in place to protect the plant and its habitat. Alternatively, efforts can be made to cultivate sea holly under controlled conditions in order to obtain its phytochemicals without contributing to the depletion of wild populations.

From a chemical composition perspective, *E. maritimum* is known to contain essential oils [5,11,23,24,25,26,27], triterpenoid saponins [6,23], flavonoids, and phenolic acids [6,7,15,23,28,29], etc. The essential oil content in the aerial parts of wild plants from Portugal [26], Corsica, Sardinia [25], and Greece [27] varied between 0.06% and 0.09%, while plants from Sicily had a much higher essential oil content at 0.93% [24]. Only one study has investigated essential oil content in the dried roots (0.01%) of *E. maritimum* of plants cultivated in a botanical garden in Poland, and it confirmed that the leaves contain more EO than the roots [5]. The dominant compounds in oils from Portugal [26] were found to be 4,4,11,11-Tetramethyl-7-tetracyclo[6.2.1.0(3.8)0(3.9)]undecanol and germacrene D. The major compounds in EO samples from Sicily [24] were found to be germacrene D and spathulenol. On the other hand, studies carried out in Poland [5] showed that the main compounds in the roots were hexadecanoic acid, menthol, and menthone. The main compounds in the leaves were 2,3,4-trimethylbenzaldehyde and germacrene D, and 2,3,4-trimethylbenzaldehyde was found in shoot culture. It is described that leaf EO components have been proven to have antifungal and antibacterial properties; for example, oxygenated sesquiterpenes 4βH-muurol-9-en-15-al and 4βH-cadin-9-en-15-ol are described to show efficiency against Listeria monocytogenes and Escherichia coli [30]. Kikowska et al. [5] found that essential oils from *E*. *maritimum* leaves were effective against *Trichophyton mentagophytes* and *Staphylococcus aureus.* Previous research [29] on Tunisian *E*. *maritimum* extracts from leaves, roots, and stems showed no significant differences in the total phenolic content (TPC) between plant parts. On the other hand, Kikowska et al. [7] reported that the total phenolic content was higher in whole plants compared to separate shoots and roots from in vitro cultures of *E. maritimum.* However, when testing fresh materials, the biomass from in vitro cultures consistently exhibited more polyphenols than the same organs from intact plants [7]. Previous studies [7,12,15,30] have demonstrated that the non-volatile chemical composition of root and leaf extracts of *E. maritimum* varies. Among the most frequently reported phytochemical compounds in *E. maritimum* samples are hydroxycinnamic acid derivatives (such as rosmarinic, chlorogenic, and caffeic acids) [8,12,13,14,30,31]. Flavonoids are also an important class of compounds with a wide range of components and are believed to contribute to the antioxidant properties of many aromatic plants used in the food, pharmaceutical, and cosmetic industries [32]. *E. maritimum* is no exception, and the total flavonoid content in its composition has been widely studied using spectrophotometric quantification methods [12,15,22,27,30]. The saponins possess amphipathic properties, which means that they have both hydrophilic and hydrophobic characteristics [33]. The first phytochemical investigations on Eryngium species’ saponin content were initiated in the early 1970s [12], and the study has continued to the present day. Saponins have been recognized by separation techniques such as thin layer chromatography (TLC) [34,35] or liquid chromatography (LC) analysis [7,17]. The identification of saponins has confirmed that Eryngium species, including *E. maritimum*, are abundant sources of triterpene saponins [6,34,35].

The aim of this study was therefore to analyze the chemical composition of both volatile and non-volatile compounds extracted from different parts of *E. maritimum* plants grown in Latvia [10], including the leaves of wild-collected specimens as well as leaves and shoots of in vitro propagated plantlets and leaves and roots of field-grown micropropagated plantlets from the same populations. It should be noted that this study represents the first time such an analysis has been conducted on this particular plant species in Latvia.

To assess phytochemical profiling, high-throughput 96-well plate spectrophotometric assays were employed to evaluate total phenolics, total sugars, total saponins, and DPPH antioxidant activity. It should be noted that the total saponin content of *E. maritimum* using spectrophotometric assays on a high-throughput 96-well plate has not been reported previously. Nonvolatile secondary metabolites were tentatively identified using liquid chromatography high-resolution mass spectrometry (LC-qTOF-MS). Additionally, the content and chemical composition of steam-distilled essential oils from wild-grown plants and acclimated plants in the field were evaluated using gas chromatography mass spectrometry (GC-MS). Our results revealed differences in the types and amounts of volatiles between the leaves and roots. Furthermore, the usefulness of headspace gas chromatography mass spectrometry (HS-GC-MS) for fast screening studies of in vitro plant volatile compounds was demonstrated. This method can be used to predict the formation of marker compounds in the same plants that have been acclimated in the field.

## 2. Results and Discussion

### 2.1. Essential Oil Quantification and Volatile Profile Characterization by GC-MS

Researchers have previously studied the chemical composition and essential oil (EO) content of numerous *Eryngium* species. Differences in essential oil content and composition between aerial parts and roots have been described previously [5,24,25].

**Figure 1:**
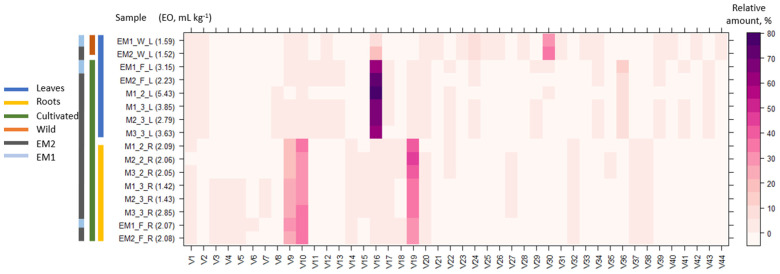
Essential oil content (mg mL^−1^) (EO) and heatmap of the chemical components of the essential oil of wild and field-grown *E. maritimum* over different samples and sample parts. The legend denotes scaled relative amounts of the volatile chemical constituents (V1–V44, according to Table 1). The deep purple color indicates higher concentrations, whereas the pale pink color indicates lower corresponding values.

Hydrodistillation of *E. maritimum* dried leaves yielded 1.52 (0.15%) to 5.43 (0.54%) mg mL^−1^ essential oil in leaves and 1.42 (0.14%) to 2.85 (0.28%) mg mL^−1^ in roots (Figure 1). The leaves from 20 wild plants (EM1 W_L and EM2 W_L) contained 1.5 to 2.0 times less essential oil than those from 20 plants grown in the agricultural field for two seasons (EM1 F_L and EM2 F_L) of the respective population. This is the first study to compare the chemical composition of wild and cultivated plants of *E*. *maritimum*. Previously, cultivation has been shown to increase the EO content in *Pelargonium inquinans* [36], decrease *Origanum syriacum* [37], and have no significant effect on EO content in *Glechoma hederaceae* [38]. The essential oil content in the leaves of a single field-grown mericlone (M1) exceeded essential oil content in the pooled sample of the wild (EM2) by 3.6 times in the first vegetation season and 1.8 times in the second vegetation season. Further extensive studies are needed to clarify whether the EO yield is influenced more by the age of the plant or the weather conditions of the growing season. The yields of essential oils obtained from the roots were lower than in leaves. In the two field-grown populations (EM1 and EM2), the essential oil yield ratio from leaves and roots was 1.1–1.5, but in single mericlones (M1, M2, and M3) in the second vegetation season it was 1.3–2.7. The essential oil yields found in wild sea holly plants (0.1%) in Latvia slightly exceed the amount of essential oil in wild collected aerial parts from Portugal (0.08%) [26], Corsica (0.06%), Sardinia (0.09%) [25], and Greece (0.09%) [27]. However, plants from Sicily were reported to have much higher essential oil content in aerial part—0.93% [24]. Such differences may arise due to genetic differences between the populations, differing environmental conditions, and biotic and abiotic stressors, as well as collection at different phenological phases. Only one study has investigated essential oil content in in dried roots (0.01%) of *E*. *maritimum* of plants cultivated in botanical garden in Poland [5], which was lower than the one found in this study. This study confirmed the previous findings that leaves contain more EO than roots [5].

Overall, 44 different volatiles (V) were identified in hydro-distilled essential oils by GC-MS (Table 1). The composition and amount of volatiles in wild and cultivated plants differed between wild and cultivated plants. Germacrene D is common among dominant compounds in both wild (7.92–18.28%) and cultivated (61.13–75.05%) plants. A previously high content of Germacrene D (10.5–40%) has been found in leaves and aerial parts in Portuguese, Italian, and Polish samples. The dominant compounds of the essential oil obtained from leaves collected in the wild were 4βH-muurol-9-en-15-al (V30) > germacrene D (V16) > spathulenol (V24) > 4βH-cadin-9-en-15-ol (V39). Similar profiles have earlier been reported in samples from Poland, Corsica, and Sardinia [5,25]. In contrast, dominant compounds in oils from Portugal [26] were 4,4,11,11-Tetramethyl-7-tetracyclo[6.2.1.0(3.8)0(3.9)]undecanol, germacrene D. The major compounds in samples from Sicily [24] were germacrene D and spathulenol, but 4βH-muurol-9-en-15-al, 4βH-cadin-9-en-15-al, 4βH-muurol-9-en-15-ol, and 4βH-cadin-9-en-15-ol were not found. The sequence of dominant compounds in leaves from field-cultivated plants was germacrene D (V16) > eudesma-4,7-diene-1β-ol (V36) > cumene (V1) > α-muurolene (V19), thus differing from the profile of wild plants. In the wild, sea holly grows in sand dunes affected by salinity from seas and oceans. In the field, soil contained more nutrients and no salinity stress, which could be a major factor contributing to the change in EO components. Previous studies [39] on *Teucrium scordium* and *Mentha pulegium* have demonstrated that the components of EOs change in adaptive response to salinity stress. Further studies on *E*. *maritimum* are needed to clarify the effect of salinity and soil fertility on EO quantitative and qualitative compositions. Dominant compounds have a major effect on the biological activity of essential oils, thus the differences in oils from various populations and differences arising from wild collection and cultivation of the species may lead to different properties and applications. The leaf EO components have been proven to have antifungal and antibacterial properties, e.g., oxygenated sesquiterpenes 4βH-muurol-9-en-15-al (V30) and 4βH-cadin-9-en-15-ol (V39) have been seen to show efficiency against *Listeria monocytogenes* and *Escherichia coli* [28]. Kikowska et al. [5] found that essential oils from *E*. *maritimum* leaves were effective against *Trichophyton mentagophytes* and *Staphylococcus aureus*. According to Machado [40], *E*. *maritimum* essential oils or some of their constituents can be potentially useful in the clinical management and research of new therapeutic agents for leishmaniasis.

The relative amounts of the *E*. *maritimum* essential oil components differed between the leaves and roots of micropropagated plants and the leaves of wild plants segregating in three distinctive clusters (Figure 1). The clusters were discriminated by a higher proportion of Germacrene D in the leaves, a higher α-muurolene, duraldehyde, and mesitaldehyde content in roots, and a higher 4βH-Muurol-9-en-15-al content in the leaves of wild plants. Differences in the composition of EO between roots and leaves have been reported in the only study previously determining the EO of both organ systems [5]. Similarly, to this study, the content of Germacrene D was more pronounced in leaves. In this study, the major compounds separated from the roots were α-muurolene (V19) > duraldehyde (V10) > mesitaldehyde (V9), but Kikowska et al. [5] reported menthol > menthone > menthyl acetate > 2,3,4-trimethylbenzaldehyde as the dominant compounds in roots, which were not detected in this study. The last one may lead to discussions because its molar mass is identical to that of duraldehyde, also referred to as 2,4,5-trimethylbenzaldehyde. A similar EO composition was observed in root samples of in vitro and field-cultivated samples. More studies on *E*. *maritimum* root EO content and composition are needed to clarify the diversity across genetical and environmental gradients.

### 2.2. Phytochemical Screening of E. maritimum Plant Extracts

The ability of *E. maritimum* aqueous ethanolic extracts to scavenge stable DPPH radicals, along with the total phenolic, saponin, and sugar contents in the extracts using 96-well plate methods, was evaluated.

Phenolic compounds are a diverse group of secondary metabolites that are often found in plants and are known to have a wide range of biological activities. The amounts of total phenolics in *E. maritimum* ethanolic extracts were estimated by the Folin–Ciocalteu method, and the obtained results varied from 0.08 to 1.02 g GAE/100 g dry weight, confirming the results of the studies published previously [7,15,27,28,30,41]. Previous studies [29] of Tunisian *E. maritimum* leaf, root, and stem extracts found no significant differences of TPC content between plant parts. According to Kikowska et al. [7], whole plants had a higher total phenolic content than separate shoots and roots from in vitro cultures of *E*. *maritimum*. However, when fresh materials were tested, the biomass from in vitro cultures always had more polyphenols than the same organs from intact plants [7]. Our results are in line with these findings. Extracts from roots showed a smaller capacity to accumulate phenolic compounds in comparison to leaves from intact plants (M(1)(2)(3)_1_L and M(1)(2)(3)_1_R). In contrast, roots from in vitro plantlets had one of the highest total phenolic content yields among all tested extracts. In contrast to previous studies, we found that polyphenols were not the dominant class of chemical compounds in *E*. *maritimum*; significantly higher amounts of total saponins were observed, varying from 2.97 to 10.27 g ESE on 100 g dry weight. To the best of our knowledge, this is the first paper presenting the 96-well plate method for determination of total saponins in *E. maritimum* leaf and root extracts by the modified anisaldehyde–sulfuric acid method. Saponins are characterized by their amphipathic nature, which means they have both hydrophilic and hydrophobic properties [33]. Beginning in the early 1970s, the first phytochemical studies of the genus *Eryngium* focused on its saponin content [12], with continuing research up to the present day confirming that not only *E*. *maritimum*, but also other *Eryngium* species are rich sources of triterpene saponins [6,34,35]. According to Kowalczyk [35], triterpenoid saponin production by *E. maritimum* in vitro root cultures was significantly lower than that of an intact plant. In contrast, in this study root and leaf samples of the same plant generated approximately the same total saponin amounts. We believe that this is because most of the previous studies were based on the identification of saponins by separation using thin layer chromatography (TLC) [34,35] and liquid chromatography (LC) analysis [7,17], while we performed total saponin testing. The presence of phenolic compounds in *E. maritimum* suggests that this plant may have potential health benefits, particularly in terms of its antioxidant and anti-inflammatory properties, including antimicrobial properties, while the unique structure of saponins characterized by a triterpene aglycone and one or more sugar chains allows them to interact with cell membranes and form complexes with cholesterol and other lipids, which can have various biological effects [33]. In accordance with studies made by Amessis-Ouchemoukh et al. [41], anti-acetylcholinesterase activity and ferrous ion-chelating power was exhibited by the methanolic extracts of *E. maritimum* leaves. Meot-Duros et al. [29] confirmed that sea holly polar fraction of leaves presented a strong antibacterial activity against two microorganisms, *Pseudomonas aeruginosa* and *P. marginalis*. A recent study demonstrated that *E*. *maritimum* rhizome aqueous extracts exhibit a rich diversity of pharmacologically bioactive compounds with potential therapeutic applications. The extract demonstrated no cytotoxicity and instead exhibited antioxidant and anti-inflammatory properties in Jurkat cells by stimulating an antioxidant response and reducing the secretion of cytokines and nitric oxide [42]. Experiments performed by Küpeli [43] on mice showed that aqueous and ethanolic extracts of the aerial parts and roots of *E. maritimum*, collected in Turkey, had strong anti-inflammatory and antinociceptive effects. Even more, the saponin–phenolic acid methanolic fractions of the Polish *E*. *maritimum* in different organs and undifferentiated in vitro culture—cell suspension—showed high antibacterial activity against *S*. *aureus* [35]. The stable DPPH radical against ascorbic acid was used within this study to assess the ability of the obtained *E. maritimum* leaf and root ethanolic extracts to scavenge radicals, suggesting the above-mentioned phytochemical group constituents’, such as phenolic compounds and saponins, capability of deactivating the free radicals. Antiradical activity (ARA) values ranged from 19.47 to 211.60 mg ASE/100 g DW, while DPPH values ranged from 3.9 to 42.5% (Table 2). According to Traversier [17], in research funded by Cosmetic Laboratoires Clarin, antiradical activity as well as biological activities such as anti-collagenase, antimicrobial, and anti-tyrosinase activity of *E. maritimum* extracts depend on the technique of extraction used. At the same time, extracts with low DPPH (%) activity (11 and 34%, respectively) showed the highest values of tyrosinase assay (123 and 130%) against kojic acid 50 μg mL^−1^ (99%) and collagenase assay (57 and 19%) in comparison with phenanthroline 2 mg mL^−1^ (132%), suggesting this plant’s potential use in different cosmetic applications. While Pereira et al. [15] described the biotechnological evaluation of the different organs of *E. maritimum* in terms of its antidiabetic and antioxidant potential in the form of decoction and tincture, DPPH scavenging of sea holly was found to be higher in extracts derived from leaves (39.1–84.2%) in comparison with roots (31.5–51.3%), confirming that these extracts may help manage type 2 diabetes and could be useful in preventing oxidative-stress-related disorders. It is also important to consider that the *E. maritimum* extracts’ total sugar content could affect their antibacterial, therapeutic, and preservation qualities. The concentration of total sugars had high variation, ranging from 0.85 to 36.85 g glucose equivalents per 100 g of dried sample. Higher concentrations of total sugars were observed in the root extracts in all cases. The antibacterial effects of concentrated sugar solutions against pathogens such as *S. aureus*, *Bacillus subtilis*, and *P. aeruginosa* have been demonstrated in earlier research [44,45]. According to Mizzi’s findings [46], high sugar concentrations inhibit bacterial growth, while very low concentrations have the opposite effect, i.e., they promote bacterial growth. This suggests that there is a critical concentration at which sugars stop acting as antimicrobial agents and start functioning as media. In very recent research by Nakurte [46], it was confirmed that total sugars extracted from *Matricaria recutita* white ray floret supercritical fluid extracts were highly positively correlated with inhibitory activity against *E. coli* and *P. aeruginosa*, with a weaker influence on inhibitory activity against *P. aeruginosa* and *S. aureus*. Plant parts, growth conditions, and population can have a significant impact on TPC, ARA, TSC, and total sugar content. The test highlighted significant differences (*p* < 0.05) between plant part and growth conditions, but no significant differences were observed between populations. Significant differences in total sugar content and TPC were observed between plant aboveground and belowground parts, whereas the growth conditions had a significant effect on TPC and ARA.

All the above-mentioned phytochemicals groups are important; therfore, we further screened and identified these bioactive compounds with LC-qTOF-MS, as it can provide more reliable and authentic data.

### 2.3. LC-MS of E. maritimum Plant Extracts

**Figure 2:**
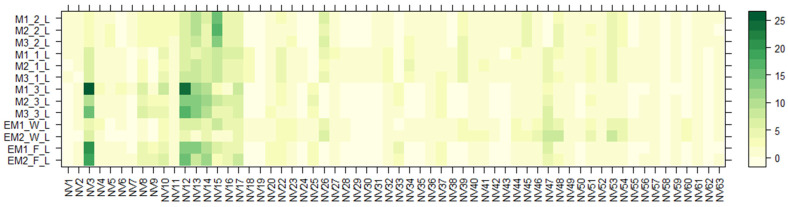
Variation in the non-volatile chemical components of the aqueous ethanolic extracts of *E. maritimum* leaves over different samples. The legend denotes scaled relative amounts (%) of the non-volatile chemical constituents (Table 3). The deep green color indicates higher concentrations, whereas the pale yellow-to-pale green color indicates lower corresponding values.

**Figure 3:**
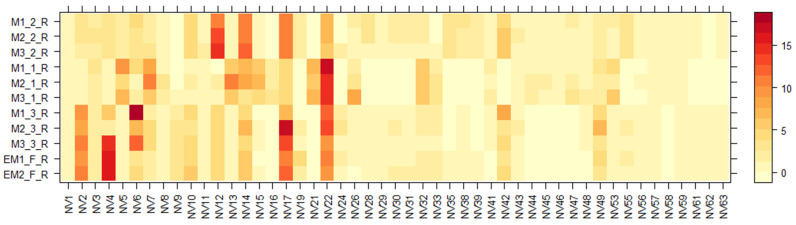
Variation in the non-volatile chemical components of the aqueous ethanolic extracts of *E. maritimum* roots over different samples. The legend denotes scaled relative amounts (%) of the non-volatile chemical constituents (Table 3). The deep red color indicates higher concentrations, whereas the pale yellow-to-orange color indicates lower corresponding values.

Using the UHPLC-ESI-q-TOF-MS technique, separation and tentative identification of nonvolatile (NV) compounds was performed on *E. maritimum* aqueous ethanolic extracts. The obtained results for all separated compounds are presented in Table 3. In total, sixty-three nonvolatile compounds (NV1-NV-63) were identified, including AA-amino acids, C-coumarins, CA-cinnamic acid derivatives, F-flavonoids, HC-hydroxycinnamic acid derivatives, O-oligosaccharides, OG-O-glycosyl compounds, PG-phenolic glycosides, QA-quinic acid derivatives, and TT-triterpenoids. As in previous studies [7,12,15,30], the non-volatile chemical compound composition of the root and leaf extracts of *E*. *maritimum* differ. Therefore, two heatmap diagrams (Figure 2 and Figure 3) were evaluated, which summarize the relative amounts (%) of all separated non-volatile compounds among all analyzed extracts.

According to Figure 2 and Figure 3, chemical composition varies between shoot and root organ systems as well as in plants obtained from different growing systems—wild, in vitro, or field conditions. One of the major groups that dominated in both leaf and root samples were amino acids (AA). A total of sixteen different amino acids and their derivatives, including seven essential AA, were detected. Several amino acids, even those that are not directly involved in making proteins, are found to play important roles in plant growth and response to changes in their environment. They are also the building blocks of many primary and secondary metabolites [47]. To determine the total yields, AA extracted from *E. maritimum* plant extracts were quantified by leucine equivalents, and their total amounts in mg per 100 g DW are summarized in Table 4. Total amino acid amounts ranged from 36.4 mg to 846.8 mg of leucine equivalents, with the highest amount released from in vitro micropropagated plant roots (M1(1); (2); (3)_R). The most dominant AA were leucine (NV12), isoleucine (NV10), phenylalanine (NV14), and tryptophan (NV17). These data are in agreement with those previously published by Taç and Özcan et al. [48], who analyzed *E. maritimum* plants located in dunes in Turkey and concluded that sea holly plants can be effectively used for biochemical and biotechnological applications in many different areas, including human nutrition and health.

The next dominant phytochemical group was hydroxycinnamic acid derivatives (HC), which include such compounds as neochlorogenic acid (NV21), chlorogenic acid (NV22), rosmarinic acid (NV49), and ferulol (NV43). This phytochemical class is one of the most often reported in *E. maritimum* samples [8,12,13,14,30,31]. The mean values of chlorogenic acid (CA) were generally higher in all our tested *E. maritimum* extracts, ranging from 15.0 mg to 5.75 g in 100 g DW, in comparison with rosmarinic acid (RA), ranging from 0.6 mg to 1.2 g in 100 g DW. However, in both cases, the most significant amounts appear in the roots of in vitro-cultured plants (M1_(1);(2);(3)_R). ANOVA revealed significant differences (*p* < 0.05) among growth conditions, while there were no significant differences between populations and plant parts. Large scale and long-term studies have been conducted to investigate the importance of RA and CA biosynthetic pathways in plants [49,50,51]; however, one thing is clear: regardless of the species, in plants these compounds can serve as one of the quality markers at every stage of plant development. According to Kikowska [6], RA and CA can be used as marker compounds to reveal how well different supplements work for *E. maritimum* micropropagation in vitro. By changing the concentration of auxins (indole-3-acetic acid (IAA), indole-3-butyric acid (IBA), or 1-naphthaleneacetic acid (NAA)) added, the concentration of RA and CA can be changed from 0.002 to 0.743 mg in 1 g DW. In the same study, similar relationships were observed: roots from in vitro-regenerated plants contain much higher yields of RA and CA compared with those from field-grown plants, while the concentration amounts between RA and CA were opposite to our study. No less important and characterized by a wide range of compounds is the class of flavonoids (F), which consists of fifteen separate compounds in total, mostly recognized as kaempferol, quercetin, luteolin, and isorhamnetin derivatives. It is believed that this compound class is among the ones contributing to antioxidant properties in a wide range aromatic plants used in the industries of food, pharmacy, and cosmetics [32]. *E. maritimum* is no exception, and the composition of flavonoids in it has been widely studied [12,15,20,27,30]. Although most of the authors performed spectrophotometric quantification of total flavonoids, finding their concentrations to be 24.3 mg quercetin equivalents (QE) in 1 g DW [41], 22 mg QE in 1 g DW [15], and 1.5 mg rutine equivalents (RE) in 1 g DW [13], estimation of total flavonoid amount using HPLC gave even lower amounts in the range of 0.29 to 0.34 mg in 1 g DW (originally 29.12 mg to 34.37 mg in 100 g DW) [7]. The observed trends are similar to our study: in the root samples they are found in a smaller number and yields compared to the leaf extracts. Because the separation and detection of triterpene saponins (TT) depend on several different parameters in the sample extraction process, as has already been said, we used spectrophotometric analysis to determine their total amount (TSC) in our study. With the help of the LC-qTOF-MS method, eryngioside derivative triterpenoids were found to be the most common saponins in the tested samples. Seven of them (NV56–NV59, NV61–NV63) were separated from *E*. *maritimum* tested extracts, but the presence of these compounds is mostly confirmed in other species, such as *E*. *yuccifolium* [52], *E*. *alpinum* [31], and *E*. *planum* [35]. According to Wang [23], eryngioside J and eryngioside L extracted from *E*. *campestre* showed moderate cytotoxic properties against different cell lines such as PANC-1, A-549, PC-3, HL-60, and MRC-5, indicating that triterpenoid saponins may provide an interesting lead for cancer drug development. 

### 2.4. Headspace-GC-MS Analysis of E. maritimum In Vitro Micropropagated Plants

Headspace gas chromatography-mass spectrometry (HS-GC-MS) analysis is a powerful technique for analyzing the volatile organic compounds (VOCs) present in fresh aromatic plants [53]. These analyses can provide a detailed profile of the volatile compounds present in fresh aromatic plants, including their chemical structures, relative concentrations, and potential bioactivities, and they can be used as fast and simple indicators of plant material authenticity. The information that is gathered can be used in several ways. For example, it can be used as a chemotaxonomic tool between in vitro micropropagated plants and their field-adapted counterparts when only a small amount of plant biomass is available, such as with in vitro samples. As far the authors know, this study is the first to describe the chemical composition of volatiles extracted from freshly grown micropropagated plant parts (shoots and roots) of *E. maritimum*. Table 5 lists the identified volatile constituents and their relative percentages in the fresh headspace specimens. The analyses allowed the identification of 33 compounds in total: 33 for the leaves and 16 for the roots (99.1% and 99.9% of the total components detected, respectively).

**Figure 4:**
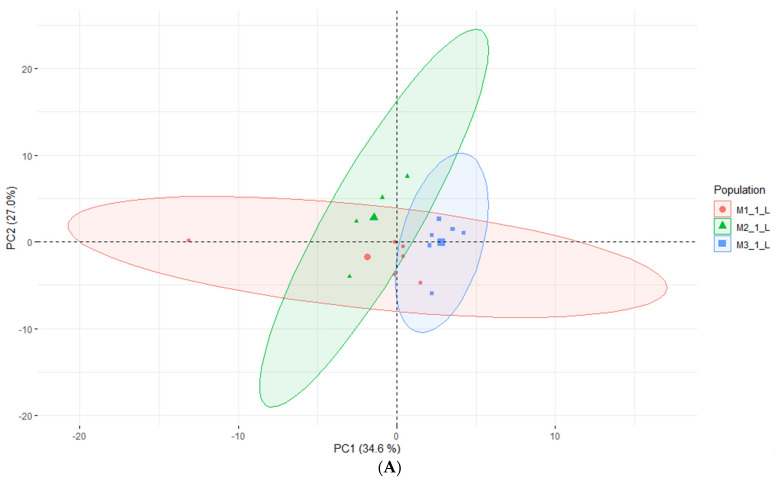

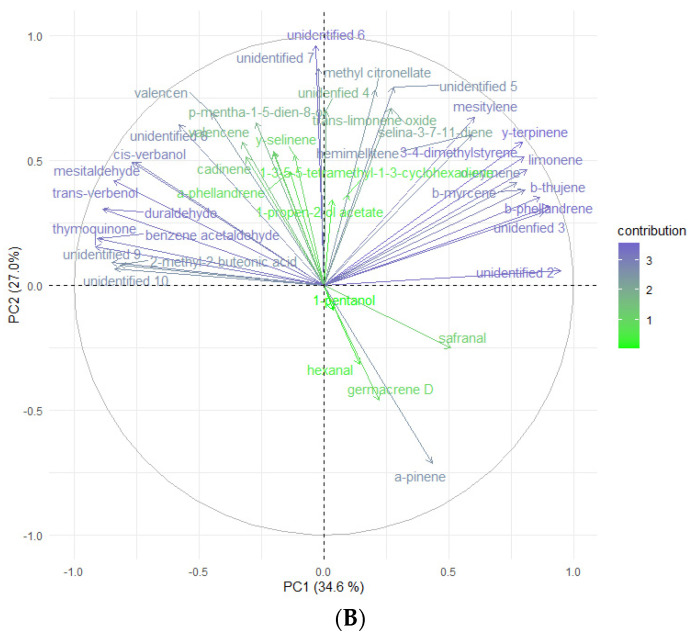
Principal component analysis (**A**) and contributions (**B**) referred to the main volatile organic compounds detected in the fresh leaves of *E*. *maritimum* in vitro samples.

**Figure 5:**
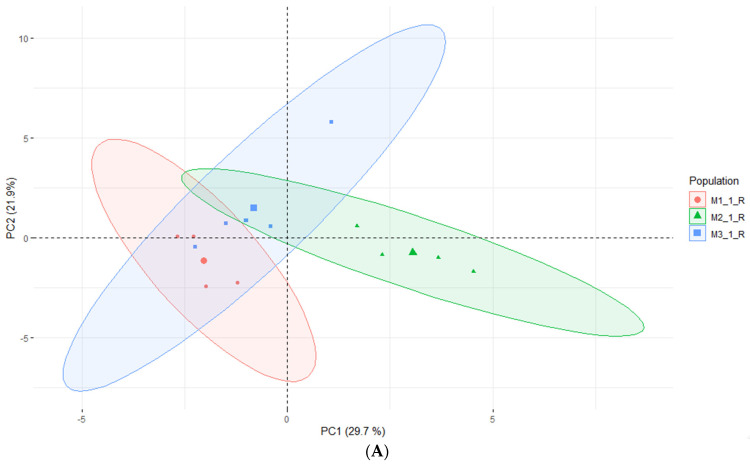

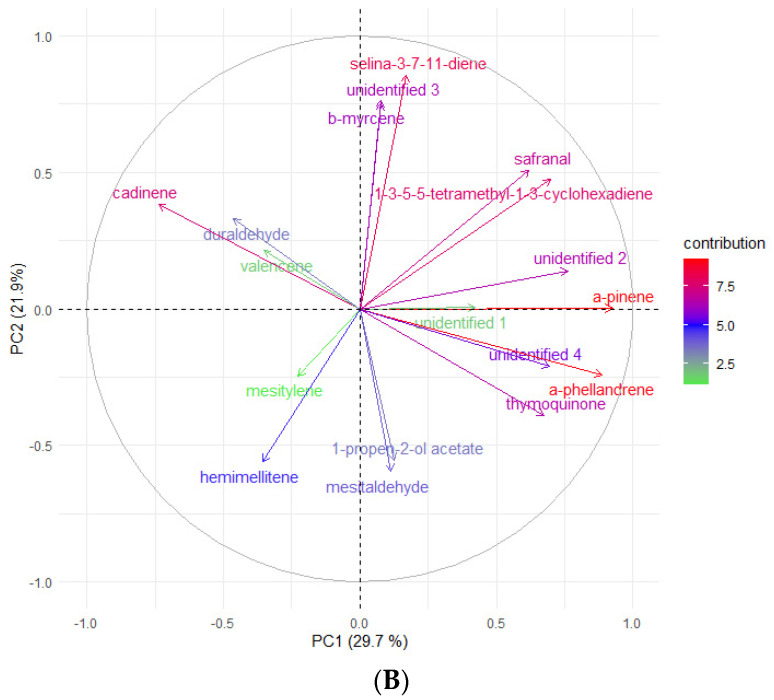
Principal component analysis (**A**) and contributions (**B**) referred to the main volatile organic compounds detected in the fresh roots of *E*. *maritimum* in vitro samples.

According to Kikowska [5], the composition of essential oils obtained from in vitro shoots and analyzed by GC-FID-MS was very similar to that of leaf oil. Most of the identified compounds were found in both oils, and the dominant constituents were 2,3,4-trimethylbenzaldehyde, followed by trans-verbenol, germacrene D, and mesytilene. As shown in Table 5, while some compounds were consistent with the EO obtained from in vitro shoots (mesytilene, trans-verbenol, germacrene D), others were found in higher concentrations in the headspace samples. The most prevalent compounds were α-pinene, mesitaldehyde, duraldehyde, valencene, β-myrcene, mesitylene, and cadinene. The most common class of chemicals, as was expected given the employed technique (static headspace), were hydrocarbon monoterpenes and sesquiterpene hydrocarbons (lower boiling points). Using the HS data, the differences between the volatile organic compounds found in fresh shoots (Figure 4) and fresh roots (Figure 5) of *E*. *maritimum* in in vitro plantlets from three mericlones were evaluated using principal component analysis (PCA). Both root and shoot samples formed clusters according to mericlones. Clusters were partially overlapping, indicating that mericlones from the same population have both common and specific volatile organic compound profiles. Mericlone M3 occupied positive values of the first principal component, but most of the samples of M2 had negative ones. The major compounds contributing to the division were β-phellandrene, β-thujene, and limonene, for the positive values, and thymoquinine, trans-verbenol, and mesitaldehyde for the negative ones. All mericlones were equally distributed along the second principal component. Regarding root samples, most of the samples of mericlone M2 had positive scores for the first principal component (Figure 5), indicating higher values of α-pinene, α-phellandrene, 1,3,5,5-tetramethyl-1,3-cyclohexadiene, and thymoquinone. PCA clustered samples of mericlone M1 on the negative scores of the first principal component, indicating higher values of cadinene, duraldehyde, valencene, mesitylene, and hemimellitene.

## 3. Materials and Methods

### 3.1. Plant Materials

The seeds and leaves of *E*. *maritimum* were gathered in September 2020 from two wild populations in Latvia, near Ziemupe (EM1) (N 56°48′4″ E 21°4′4″) and Užava (EM2) (N 57°14′49″ E 21°25′52″). Permits for the collection of protected plants were obtained from the Nature Conservation Agency (decision no. 3.6/470/2020-N5 issued on 13 August 2020). The leaves of the 20 randomly sampled wild plants were used for chemical analysis (Table 6). Collected seeds were used for plant propagation in vitro as previously described [10]. The shoots and roots of micropropagated plantlets of three mericlones (M1, M2, and M3) of the population EM2 were subjected to chemical analysis. Micropropagated plantlets were adapted to ex vitro conditions for 60 days and further planted in field conditions in June 2021 and cultivated as previously described [10]. Roots and leaves were collected from the plantlets grown in field conditions for three months (September 2021) and at the next vegetation season (September 2022). Twenty plantlets each from different mericlone of populations EM1 and EM2 were sampled after three-month cultivation under field conditions. Three mericlones (M1, M2, and M3) of the population EM2 were sampled both after three-month cultivation and at the next vegetation season.

### 3.2. Chemicals and Reagents

The process of preparing the extract involved the use of ethanol (96%) sourced from Kalsnavas Elevators Ltd. (Jaunkalsnava, Latvia). LC-MS grade acetonitrile, methanol, and formic acid were procured from Fisher Scientific (Loughborough, UK), while water used for LC-qTOF-MS analysis was purified via a Smart2Pure water purification system (Thermo Scientific, Dreieich, Germany). Gallic acid, cyclohexane, xylene, ascorbic acid, and chlorogenic acid were obtained from Acros Organics (Geel, Belgium), and Na_2_CO_3_ was sourced from Honeywell (Charlotte, NC, USA). In addition, 2,2-diphenyl-1-picrylhydrazyl (DPPH) and phenol were purchased from Alfa Aesar (Kandel, Germany). We used Folin–Ciocalteu reagent, H_2_SO_4_, HNO_3_, HCl, and D-glucose, all of which were purchased from Fisher Scientific (Loughborough, UK). Standards of rosmarinic acid and leucine were obtained from Sigma-Aldrich (St. Louis, MO, USA).

### 3.3. Essential Oil Quantification and Volatile Profile Characterization by GC-MS

The plant samples were dried at 38 °C for analysis. The essential oils were prepared using a Clevenger-type hydrodistillation apparatus. In brief, 10 grams of powdered herbal drug were placed in a 500 mL flask, distilled water was added as the distillation liquid, and 0.50 mL of xylene was added to a graduated tube. The distillation process was carried out at a rate of 3–4 mL min^−1^ for 3 hours. The essential oil yield (mL kg^−1^) was calculated based on the dried weight of the samples. The oil was then separated and dried over anhydrous sodium sulfate to remove any moisture and preserved in a sealed amber glass vial at 4 °C until GC–MS analysis. For the volatile profile characterization of the essential oils, samples were diluted in cyclohexane, mixed, and injected into a GC-MS system. The analyses were conducted using an Agilent Technologies 7820A gas chromatograph equipped with an Agilent 5977B mass selective detector (MSD). The system utilized a non-polar HP-5 capillary column (60 m × 0.25 mm, 0.25 µm film thickness) coated with 5% phenyl and 95% methyl polysiloxane. Helium (He) was used as the carrier gas with a split ratio of 1:100 and a flow rate of 1.5 mL/min. A volume of 3 μL was injected, and the temperature program started at 70 °C and was then increased at a rate of 5 °C/min to 230 °C, followed by an increase to 295 °C at a rate of 7 °C/min. Finally, the temperature was maintained at 295 °C for 30 min, and the injector temperature was set at 270 °C. The mass spectra were recorded at 70 eV within a range of 70–500 *m*/*z*. The ion source temperature was maintained at 230 °C. The components were identified based on their retention indices, determined with reference to a homologous series of C5–C24 n-alkanes, and by comparison of their mass spectra with those stored in the National Institute of Standards and Technology (NIST) MS Search 2.2 library. The GC-MS data were analyzed using the Agilent MassHunter Qualitative Analysis 10.0 data acquisition software. The content of separated compounds was calculated in peak areas using the normalization method without correction factors (reported compound contents are only approximate without the determination of response factors).

### 3.4. The Identification and Quantification of Volatile Compounds by HS-GC-MS Analysis

Fresh sea holly leaves and roots were collected from experimental plants and were placed into 20 mL headspace vials. Analysis was carried out using a Gerstel MPS autosampler in conjunction with an Agilent Technologies 7820A gas chromatograph and Agilent 5977B mass selective detector (MSD). A polar CP-Wax 52CB capillary column (50 m × 0.32 mm, 0.20 µm film thickness) was used. The carrier gas was helium (He) with a split ratio of 1:50 and a flow rate of 1.0 mL min^−1^. The incubation temperature was 100 °C, the incubation period was 25 min, the agitator cycle was 30 s on and 15 s off, and the injection volume was 1700 μL. The temperature program was started at 60 °C and was then increased at a rate of 7 °C min^−1^ to 250 °C, which was then maintained for 2 min. The temperature of the injector was 250 °C. The mass spectra were recorded at 70 eV. The mass range was 50–650 *m*/*z*. The ion source temperature was maintained at 230 °C. The components were identified on the basis of their retention indices (calculated with reference to the homologous series of C5–C24 n-alkanes) by comparing their mass spectra to those in the National Institute of Standards and Technology (NIST) MS Search 2.2 library. To analyze GC-MS data, the Agilent MassHunter Qualitative Analysis 10.0 data program was utilized. The amount of separated compounds was calculated in peak areas using the normalization method without correction factors (reported compound contents are only approximate without the determination of response factors).

### 3.5. Extract Preparation for UHPLC-HRMS Analysis and 96-Well Plate Assays

For all studied samples (Table 6), aqueous ethanolic extracts of dried samples (leaves and roots) were prepared by dissolving the samples in 70% ethanol at a ratio of 1:10. Samples were mixed and heated at 70 °C for 60 min in an ultrasound bath. The samples were cooled and centrifuged for 10 min at 4400 rpm. The obtained solutions were filtered through a membrane filter with pores of 0.45 µm and diluted to an appropriate concentration for analysis.

### 3.6. UHPLC-HRMS Analysis

Using an Agilent 1290 Infinity II series HPLC system combined with an Agilent 6530 qTOF MS system, the undiluted extracts were analyzed. A Zorbax Eclipse Plus C18 Rapid Resolution HD column with a flow rate of 0.3 mL/min and column oven temperature of 50 °C was utilized. The sample injection volume was 3 μL, and the needle was washed for 30 s with 70% methanol. The mobile phase consisted of a combination of A (0.1% formic acid in water) and B (0.1% formic acid in acetonitrile), using a gradient elution program. This is how the gradient elution program was implemented: Initial 2%B, 0–2 min 2%B, 2–10 min 40%B, 10–20 min 80%B, 20–27 min 95%B, 27–40 min 95%B, 40–42 min 1%B. At 280 nm and 330 nm, the UV/Vis spectra were captured. The mass spectrometer was configured for 70 V fragmentation, 325 °C gas temperature, 10 L min^−1^ drying gas, 20 psi nebulizer pressure, 400 °C sheath gas temperature, and 12 L min^−1^ sheath gas flow. The source was electrospray ionization in positive mode. Mass spectra in the *m*/*z* range of 50 to 2000 were obtained using internal reference masses of 121.050873 *m*/*z* and 922.009798 *m*/*z* (G1969-85001 ESI-TOF Reference Mass Solution Kit, Agilent Technologies & Supelco). Using the Agilent MassHunter Qualitative Analysis 10.0 data acquisition software, the LC-MS data were analyzed. For the identification of isolated compounds, the Agilent MassHunter Metlin Metabolomics Database and LipidMaps Database were used. For quantification of individual compounds and classes, specific individual standards were prepared according to Table 7.

### 3.7. 96-Well Plate Assays

Samples were diluted with ethanol (96%) to obtain a minimum concentration of 10 mg mL^−1^. The amounts of total phenolic content (TPC), total sugars, and antiradical activity in the examined extracts were determined using an Epoch2 UV/VIS Microplate Spectrophotometer (BioTek, Agilent, Germany), in triplicate, as previously described by Nakurte et al. [46]. A modified anisaldehyde–sulfuric acid method was used to determine total saponin content (TSC) [54]. The experimental procedure is briefly described in Table 8.

### 3.8. Statistical Analysis

Levelplots, PCA, and one-way ANOVA test with scaled or non-scaled data were created in the R software version 4.2.3. The levelplots were createdusing the package “lattice”, while PCA was created with the package “factoMineR”. A one-way ANOVA test, followed by Tukey’s test (*p* < 0.05), was used to analyze the impact of growth condition, population, or plant part on the TPC, TSC, total sugar content, and ARA.

## 4. Conclusions

Considering the endangered status of *E. maritimum*, it is crucial to practice sustainable harvesting with appropriate regulations and permits in place to safeguard the plant and its ecosystem. Alternatively, the cultivation of sea holly under controlled conditions can be pursued to obtain its phytochemicals without compromising wild populations. In this regard, it is essential to conduct a comprehensive investigation of the chemical profile of the cultivated plants. The insights gained from such studies can be of immense value to the industry. During the implementation of this research, we successfully accomplished our objectives, which included a comparative analysis of the chemical composition of leaves from *Eryngium maritimum* wild-grown plants of Latvian origin, as well as the shoots and roots of micropropagated plantlets and the shoots and roots of field-grown plants. Essential oils were extracted from the plants by hydrodistillation. The results showed that leaves from wild plants contained 1.5–2.0 times less essential oil than plants grown in an agricultural field. Hydro-distilled essential oils were analyzed using GC-MS, and 44 distinct volatiles were detected. The predominant compounds found in both wild and cultivated samples were identified as 4βH-muurol-9-en-15-al, germacrene D, spathulenol, and 4βH-cadin-9-ent-15-ol. Forty-four different volatiles were identified in hydro-distilled essential oils by GC-MS, with the most common compounds being within wild and cultivated samples, these being 4βH-muurol-9-en-15-al, germacrene D, spathulenol, and 4βH-cadin-9-ent-15-ol. The aqueous ethanol extracts were evaluated for their ability to scavenge stable DPPH radicals and for their total phenolic, saponin, and sugar contents by high-throughput 96-well plate spectrophotometric assays. The results showed that the root and leaf extracts contained different chemical compositions, with leaves containing more phenolic compounds and roots containing more sugars. Triterpenoid saponin production by *E. maritimum* in in vitro root cultures was significantly lower than that of an intact plant, but both root and leaf samples of the same plant generated approximately the same total saponin amounts. This suggests that this plant may have potential health benefits, particularly in terms of its antioxidant and anti-inflammatory properties, including antimicrobial properties. Additionally, nonvolatile compounds were separated and analyzed using UHPLC-ESI-q-TOF-MS, revealing a total of sixty-three compounds, including amino acids, hydroxycinnamic acid derivatives, flavonoids, and others. The chemical composition was found to vary between the shoot and root organ systems as well as in plants obtained from different growing systems. Amino acids were found to be the most dominant group, with leucine, isoleucine, phenylalanine, and tryptophan being the most abundant; thus, the species has potential as a novel food plant as a source of essential amino acids. Hydroxycinnamic acid derivatives were also prominent, with chlorogenic acid being the most abundant compound. Flavonoids, consisting mostly of kaempferol, quercetin, luteolin, and isorhamnetin derivatives, were also identified. Even more, this study is the first to describe the chemical composition of volatiles extracted from the freshly grown micropropagated plant parts (shoots and roots) of *E. maritimum* using headspace gas chromatography-mass spectrometry. The abovementioned findings suggest that *E. maritimum* plants can be used for biochemical and biotechnological purposes in a variety of fields, including human nutrition and health.

## Figures and Tables

**Table 1:** Volatile compound composition (%) in the essential oils made from the dried leaves and roots of wild and cultivated *E. maritimum* plants.

No	RI ^a^	Compound ^b^	Composition Range (Leaves)	Composition Range (Roots)
V1	921	Cumene	0.18–5.15	n.d–2.39
V2	937	α-Pinene	0.60–4.23	n.d
V3	972	Mesitylene	n.d	n.d–0.83
V4	990	Pseudocumene	n.d	n.d–0.41
V5	1201	Safranal	n.d	n.d–0.43
V6	1283	α-Terpinen-7-al	n.d	n.d–0.19
V7	956	Dehydrosabinene	n.d	n.d–0.28
V8	1293	Dihydroedulan	0.61	n.d
V9	1337	Mesitaldehyde	n.d–1.57	16.15–27.30
V10	1364	Duraldehyde	1.03–2.59	27.62–34.16
V11	1376	α-Copaene	n.d–1.09	n.d
V12	1384	α-Bourbonene	n.d–1.18	n.d
V13	1448	Isogermacrene D	n.d–0.72	n.d
V14	1375	Isoledene	n.d	0.56–1.31
V15	1464	dehydro-Aromadendrene	n.d	n.d–0.42
V16	1481	Germacrene D	17.92–75.05	0.39–2.55
V17	1409	α-Gurjunene	n.d–1.14	0.12–0.88
V18	1492	Valencene	n.d	n.d–1.05
V19	1499	α-Muurolene	n.d–4.85	29.64–46.83
V20	1481	Cadina-1(6),4-diene	0.72–1.21	n.d–1.10
V21	1506	α-Farnesene	n.d–1.67	n.d
V22	1645	δ-Cadinol	n.d–1.55	n.d–1.04
V23	1573	1,5-epoxysalvial-4(14)-ene	n.d–2.29	n.d
V24	1576	Spathulenol	n.d–8.02	n.d
V25	1581	Caryophyllene oxide	n.d–3.45	n.d
V26	1595	Mint ketone	n.d–3.09	n.d
V27	1490	β-Guaiene	n.d	n.d–0.45
V28	1440	Aromadendrane	n.d–0.91	n.d
V29	1631	Ledene oxide-(II)	n.d–2.28	n.d
V30	1695	4βH-Muurol-9-en-15-al	n.d–33.79	n.d
V31	1619	Patchoulane	n.d–1.02	n.d
V32	1635	β-Vatirenene	n.d	0.33–1.12
V33	1778	Isovalencenol	n.d–0.88	n.d
V34	1648	Alloaromadendrene oxide-1	n.d–2.48	n.d
V35	1640	tau-Cadinol	n.d	n.d–0.36
V36	1671	Eudesma-4,7-diene-1β-ol	n.d–11.95	n.d
V37	1712	(E)-γ-Atlantone	n.d	0.3–1.11
V38	2040	Falcarinol	n.d	0.11–0.66
V39	1742	4βH-Cadin-9-en-15-ol	n.d–5.14	n.d
V40	1876	Murolan-3,9(11)-diene-10-peroxy	n.d–1.32	n.d
V41	1762	Methyl farnesate	n.d–0.88	n.d
V42	1777	15-hydroxy-α-muurolene	n.d–1.49	n.d
V43	1837	Neophytadiene	n.d–2.50	n.d
V44	1844	6,10,14-trimethyl-2-Pentadecanone	n.d–0.59	n.d
Yields, mL kg^−1^			1.52–5.43	1.42–2.85

^a^ Experimental retention indexes (RI) were determined using the HP-5MS capillary column and was found to be consistent with values reported in the literature. ^b^ Based on a National Institute of Standards and Technology (NIST) MS Search 2.2 library. n.d—not detected.

**Table 2:** Content of total phenolics, saponins, sugars, and DPPH free radical scavenging activity of different *E. maritimum* ethanolic extracts.

Sample Code	TPC ^a^, g GAE/100 g	TSC ^b^, g ESC/100 g	Sugars ^c^, g GLE/100 g	ARA ^d^, mg ASE/100 g	DPPH ^e^ Quenched, %	IC50, µg/mL
EM1 W_L	0.64 ± 0.10	8.39 ± 0.21	5.91 ± 0.23	87.20 ± 1.26	17.5 ± 0.69	43.76 ± 1.51
EM2 W_L	0.46 ± 0.03	6.51 ± 0.16	2.49 ± 0.11	78.90 ± 1.14	15.8 ± 0.63	39.60 ± 1.38
EM1 F_L	0.83 ± 0.07	3.95 ± 0.19	2.63 ± 0.11	57.58 ± 0.83	11.6 ± 0.46	28.89 ± 0.99
EM2 F_L	0.77 ± 0.05	6.04 ± 0.25	2.22 ± 0.09	54.41 ± 0.78	10.9 ± 0.43	27.31 ± 0.94
EM1 F_R	0.22 ± 0.02	6.77 ± 0.30	12.76 ± 0.48	19.47 ± 0.52	3.9 ± 0.21	9.77 ± 0.36
EM2 F_R	0.19 ± 0.01	6.25 ± 0.20	12.68 ± 0.48	24.81 ± 0.60	5.0 ± 0.27	12.45 ± 0.46
M1_1_L	0.91 ± 0.12	6.64 ± 0.22	0.91 ± 0.05	115.20 ± 1.63	23.1 ± 0.88	57.81 ± 1.96
M2_1_L	0.85 ± 0.08	6.76 ± 0.32	0.85 ± 0.05	102.00 ± 1.47	20.5 ± 0.78	51.19 ± 1.74
M3_1_L	0.91 ± 0.09	8.27 ± 0.36	1.58 ± 0.07	117.30 ± 1.67	23.5 ± 0.90	58.87 ± 2.00
M1_1_R	0.97 ± 0.08	8.22 ± 0.48	16.56 ± 0.50	211.60 ± 2.99	42.5 ± 1.61	106.19 ± 3.51
M2_1_R	0.94 ± 0.07	10.27 ± 0.44	16.62 ± 0.47	202.90 ± 2.88	40.7 ± 1.54	101.83 ± 3.34
M3_1_R	0.80 ± 0.06	9.62 ± 0.34	15.48 ± 0.51	201.60 ± 2.73	40.5 ± 1.53	101.17 ± 3.32
M1_2_L	0.90 ± 0.07	8.64 ± 0.46	6.38 ± 0.24	179.18 ± 2.54	36.0 ± 1.37	89.92 ± 2.93
M2_2_L	1.02 ± 0.11	10.07 ± 0.42	6.93 ± 0.26	183.74 ± 2.61	36.9 ± 1.41	92.21 ± 3.01
M3_2_L	0.89 ± 0.05	9.39 ± 0.10	5.84 ± 0.22	170.33 ± 2.41	34.2 ± 1.29	85.48 ± 2.74
M1_2_R	0.08 ± 0.01	2.97 ± 0.12	21.85 ± 0.65	34.24 ± 0.49	6.9 ± 0.29	17.18 ± 0.64
M2_2_R	0.10 ± 0.02	3.26 ± 0.15	30.08 ± 0.78	38.12 ± 0.55	7.7 ± 0.33	19.13 ± 0.71
M3_2_R	0.11 ± 0.02	3.14 ± 0.22	30.36 ± 0.87	41.79 ± 0.60	8.4 ± 0.36	20.97 ± 0.78
M1_3_L	0.58 ± 0.07	6.94 ± 0.24	4.06 ± 0.34	64.79 ± 0.93	13.0 ± 0.53	32.52 ± 1.15
M2_3_L	0.59 ± 0.05	7.18 ± 0.27	6.04 ± 0.36	66.95 ± 0.96	13.4 ± 0.55	33.60 ± 1.21
M3_3_L	0.60 ± 0.03	6.98 ± 0.18	5.45 ± 0.27	69.81 ± 1.00	14.0 ± 0.59	35.04 ± 1.26
M1_3_R	0.31 ± 0.02	5.53 ± 0.20	27.67 ± 0.67	41.62 ± 0.60	8.4 ± 0.36	20.89 ± 0.78
M2_3_R	0.37 ± 0.04	6.12 ± 0.33	36.85 ± 0.54	50.15 ± 0.72	10.1 ± 0.43	25.17 ± 0.93
M3_3_R	0.27 ± 0.06	4.54 ± 0.25	26.02 ± 0.40	48.14 ± 0.70	9.7 ± 0.41	24.16 ± 0.89

^a^ Total phenolic content is expressed as the gallic acid equivalents per 100 g of dry weight (g GAE/100 g). ^b^ Total saponin content is expressed as the escin equivalents per 100 g of dry weight (g ESE/100 g). ^c^ Total sugar content is expressed as the glucose equivalents per 100 g of dry weight (g GLE/100 g). ^d^ ARA radical scavenging activity is expressed as the ascorbic acid equivalents per 100 g of dry weight (mg ASE/100 g). ^e^ DPPH radical scavenging activity of 10% extracts (on a dry basis) expressed in %.

**Table 3:** Tentative phytocomponents, extracted from the aqueous ethanol extracts of *E. maritimum* dried leaves and roots, detected by LC-qTOF-MS.

No.	RT (min)	Proposed Compound	Tentative Molecular Formula	Ion Mode	Theoretical (*m*/*z*)	Observed (*m*/*z*)	Mass Error (ppm)	Class ^a^
NV1	1.34	Histidine	C_6_H_9_N_3_O_2_	[M+H]^+^	156.0768	156.0764	0.4	AA
NV2	1.36	Arginine	C_6_H_14_N_4_O_2_	[M+H]^+^	175.119	175.1156	3.4	AA
NV3	1.58	Sucrose	C_12_H_22_O_11_	[M+K]^+^	381.0794	381.0803	0.9	O
NV4	1.61	Proline	C_5_H_9_NO_2_	[M+H]^+^	116.0702	116.0695	0.7	AA
NV5	1.62	Methionine	C_5_H_11_NO_2_S	[M+H]^+^	150.0583	150.0572	1.1	AA
NV6	1.64	Glutamine	C_5_H_10_N_2_O_3_	[M+H]^+^	147.0764	147.0772	0.8	AA
NV7	1.65	Asparagine	C_4_H_8_N_2_O_3_	[M+H]^+^	133.0608	133.059	1.8	AA
NV8	1.72	N-(1-deoxy-D-fructos-1-yl)-L-Valine	C_11_H_21_NO_7_	[M+H]^+^	280.1391	280.1382	0.9	AA
NV9	1.80	Valine	C_5_H_11_NO_2_	[M+H]^+^	118.0863	118.0848	1.5	AA
NV10	1.82	Isoleucine	C_6_H_13_NO_2_	[M+H]^+^	132.1019	132.1021	0.2	AA
NV11	2.18	Tyrosine	C_9_H_11_NO_3_	[M+H]^+^	182.0812	182.0797	1.5	AA
NV12	2.29	Leucine	C_6_H_13_NO_2_	[M+H]^+^	132.1019	132.1006	1.3	AA
NV13	2.42	N-(1-deoxy-D-fructos-1-yl)-L-Leucine	C_12_H_23_NO_7_	[M+H]^+^	294.1562	294.1573	1.1	AA
NV14	3.84	Phenylalanine	C_9_H_11_NO_2_	[M+H]^+^	166.0863	166.085	1.3	AA
NV15	3.96	N-(1-deoxy-D-fructos-1-yl)-L-Phenylalanine	C_15_H_21_NO_7_	[M+H]^+^	328.1503	328.1516	1.3	AA
NV16	5.07	N-(1-deoxy-D-fructos-1-yl)-L-Tryptophan	C_17_H_22_N_2_O_7_	[M+H]^+^	367.1538	367.1521	1.7	AA
NV17	6.35	Tryptophan	C_11_H_12_N_2_O_2_	[M+H]^+^	205.0972	205.0983	1.1	AA
NV18	6.96	7-methoxycoumarin	C_10_H_8_O_3_	[M+H]^+^	177.0546	177.0552	0.6	C
NV19	7.00	Methyl cinnamate	C_10_H_10_O_2_	[M+H]^+^	163.0754	163.0756	0.2	CA
NV20	7.63	Caffeic acid 3-O-glucuronide	C_15_H_16_O_10_	[M+K]^+^	379.0636	379.064	0.4	CA
NV21	7.71	Neochlorogenic acid	C_16_H_18_O_9_	[M+H]^+^	355.1024	355.1052	2.8	HC
NV22	7.88	Chlorogenic acid	C_16_H_18_O_9_	[M+H]^+^	355.1024	355.1056	3.2	HC
NV23	7.99	p-Coumaric acid 4-O-glucoside	C_15_H_18_O_8_	[M+NH_4_]^+^	344.1340	344.1379	3.9	PG
NV24	8.41	alpha-L-Fucopyranosyl-(1->2)-beta-D-galactopyranosyl-(1->4)-D-glucose isomer I	C_18_H_32_O_15_	[M+H]^+^	489.1814	489.1799	1.5	O
NV25	8.56	Unknown compound	-	-	-	409.185	-	-
NV26	9.19	Kaempferol 3-O-(6″-malonyl-glucoside)	C_24_H_22_O_14_	[M+H]^+^	535.1082	535.114	5.8	F
NV27	9.25	n-p-Coumaroylquinic acid	C_16_H_18_O_8_	[M+H]^+^	339.1074	339.1085	1.1	QA
NV28	9.49	alpha-L-Fucopyranosyl-(1->2)-beta-D-galactopyranosyl-(1->4)-D-glucose isomer II	C_18_H_32_O_15_	[M+H]^+^	489.1814	489.1794	2.0	O
NV29	9.97	Phenolic glycoside	C_16_H_22_O_8_	[M+Na]^+^	365.1207	365.1215	0.8	PG
NV30	9.98	Phenolic glycoside I	C_16_H_24_O_7_	[M+H]^+^	329.1595	329.1614	1.9	PG
NV31	9.99	Phenolic glycoside II	C_16_H_24_O_7_	[M+H]^+^	329.1595	329.1617	2.2	PG
NV32	9.56	n-Feruloylquinic acid I	C_17_H_20_O_9_	[M+H]^+^	369.118	369.1186	0.6	QA
NV33	10.01	n-Feruloylquinic acid II	C_17_H_20_O_9_	[M+H]^+^	369.118	369.1188	0.8	QA
NV34	10.10	8-Epiiridodial glucoside	C_16_H_26_O_7_	[M+NH_4_]^+^	348.2017	348.2047	3.0	OG
NV35	10.75	Phenolic glycoside	C_19_H_26_O_10_	[M+NH_4_]^+^	432.1864	432.1892	2.8	QA
NV36	11.43	Quercetin 3-O-galactoside 7-O-rhamnoside	C_27_H_30_O_16_	[M+H]^+^	611.1607	611.162	1.3	F
NV37	11.56	alpha-L-Rhamnopyranosyl-(1->3)-alpha-D-galactopyranosyl-(1->3)-L-fucose	C_18_H_32_O_14_	[M+H]^+^	495.1684	495.1872	18.8	O
NV38	11.94	Unknown compound	-	-	-	575.179	-	-
NV39	11.95	Phenylethyl primeveroside	C_19_H_28_O_10_	[M+Na]^+^	439.1575	439.1602	2.7	OG
NV40	11.97	Quercetin 3-O-glucoside	C_21_H_20_O_12_	[M+H]^+^	465.1028	465.1044	1.6	F
NV41	12.18	Quercetin 3-O-(6″-malonyl-glucoside)	C_24_H_22_O_15_	[M+H]^+^	551.1031	551.1047	1.6	F
NV42	12.57	Phenolic glycoside	C_19_H_26_O_10_	[M+H]^+^	415.1599	415.1671	7.2	PG
NV43	12.64	Ferulol	C_10_H_14_O_2_	[M+H]^+^	167.1067	167.1063	0.4	HC
NV44	12.76	Luteolin 7-O-rutinoside/Kaempferol 3-O-galactoside	C_27_H_30_O_15_	[M+H]^+^	595.1657	595.1679	2.2	F
NV45	12.77	Luteolin 6-C-glucoside/Kaempferol 3-O-galactoside	C_21_H_20_O_11_	[M+H]^+^	449.1078	449.1097	1.9	F
NV46	13.18	Isorhamnetin 3-O-glucoside 7-O-rhamnoside	C_28_H_32_O_16_	[M+H]^+^	625.1763	625.1797	3.4	F
NV47	13.37	Luteolin 6-C-glucoside/Kaempferol 3-O-galactoside	C_21_H_20_O_11_	[M+H]^+^	449.1078	449.1106	2.8	F
NV48	13.80	Isorhamnetin 3-O-glucoside	C_22_H_22_O_12_	[M+H]^+^	479.1184	479.119	0.6	F
NV49	13.84	Rosmarinic acid	C_18_H_16_O_8_	[M+Na]^+^	383.0737	383.0732	0.5	HC
NV50	14.25	Kaempferol 3-O-arabinoside	C_20_H_18_O_10_	[M+H]^+^	419.0973	419.0993	2.0	F
NV51	14.55	6″-Malonylastragalin	C_24_H_22_O_14_	[M+H]^+^	535.1082	535.1089	0.7	F
NV52	14.92	Kaempferol 3-O-alpha-L-rhamnofuranoside	C_21_H_20_O_10_	[M+H]^+^	433.1129	433.1144	1.5	F
NV53	15.35	Kaempferol 3-O-alpha-L-arabinopyranosyl-7-O-alpha-L-rhamnopyranoside	C_26_H_28_O_14_	[M+H]^+^	565.1552	565.1537	1.5	F
NV54	15.87	Kaempferol 3-(6-acetylgalactoside)	C_23_H_22_O_12_	[M+H]^+^	491.1184	491.121	2.6	F
NV55	16.07	Unknown compound	-	-	-	341.1419	-	-
NV56	21.28	Eryngioside F/Eryngioside H/Eryngioside I	C_52_H_82_O_21_	[M+Na]^+^	1065.5241	1065.5248	0.7	TT
NV57	21.98	Eryngioside J	C_54_H_84_O_23_	[M+Na]^+^	1123.5296	1123.5297	0.1	TT
NV58	22.42	Eryngioside C	C_54_H_88_O_24_	[M+Na]^+^	1143.5558	1143.5556	0.2	TT
NV59	23.35	Eryngioside F/Eryngioside H/Eryngioside I	C_52_H_82_O_21_	[M+Na]^+^	1065.5241	1065.5244	0.3	TT
NV60	24.09	6-Hydroxykaempferol 3,6,7-triglucoside or other kaempferol triglucoside	C_33_H_40_O_22_	[M+H-H_2_O]^+^	771.1984	771.198	0.4	F
NV61	24.67	Eryngioside K/Eryngioside L	C_54_H_84_O_22_	[M+Na]^+^	1107.5346	1107.537	2.4	TT
NV62	24.67	Eryngioside K/Eryngioside L	C_54_H_84_O_22_	[M+Na]^+^	1107.5346	1107.5374	2.8	TT
NV63	25.12	Eryngioside F/Eryngioside H/Eryngioside I	C_52_H_82_O_21_	[M+Na]^+^	1065.5241	1065.5243	0.2	TT

^a^ Group of compounds: AA—amino acids, C—coumarins, CA—cinnamic acid derivatives, F—flavonoids, HC—hydroxycinnamic acid derivatives, O—oligosaccharides, OG—O-glycosyl compounds, PG—phenolic glycosides, QA—quinic acid derivatives, TT—triterpenoid.

**Table 4:** The content of total amino acids and two selected phenolic acids in the aqueous ethanolic extracts obtained from *E. maritimum* leaf and root samples.

Sample	Amino Acids (AA), mg/100 g DW	Chlorogenic Acid (CA), mg/100 g DW	Rosmarinic Acid (RA), mg/100 g DW
EM1 W_L	53.3 ± 1.6	106.0 ± 1.7	2.8 ± 0.4
EM2 W_L	29.5 ± 0.9	78.0 ± 2.1	2.3 ± 0.3
EM1 F_L	49.5 ± 1.5	51.0 ± 1.1	0.9 ± 0.1
EM2 F_L	67.9 ± 2.1	29.0 ± 0.6	0.9 ± 0.1
EM1 F_R	36.5 ± 1.1	51.0 ± 1.2	4.0 ± 0.5
EM2 F_R	38.1 ± 1.2	40.0 ± 0.9	2.3 ± 0.3
M1_1_L	184.0 ± 4.3	120.0 ± 2.0	259.0 ± 3.2
M2_1_L	187.6 ± 4.4	72.0 ± 1.2	181.0 ± 2.3
M3_1_L	180.5 ± 4.3	87.0 ± 1.5	266.0 ± 3.3
M1_1_R	414.2 ± 9.8	2360.0 ± 2.9	674.0 ± 8.4
M2_1_R	529.4 ± 12.6	2560.0 ± 2.8	752.0 ± 9.4
M3_1_R	846.8 ± 20.1	5750.0 ± 2.7	1202.0 ± 15.9
M1_2_L	112.6 ± 2.7	159.0 ± 2.3	3.2 ± 0.4
M2_2_L	113.1 ± 2.7	180.0 ± 1.8	4.8 ± 0.6
M3_2_L	108.4 ± 2.6	151.0 ± 2.0	4.9 ± 0.5
M1_2_R	63.1 ± 1.5	61.0 ± 0.8	3.1 ± 0.4
M2_2_R	66.0 ± 1.6	75.0 ± 1.2	3.7 ± 0.5
M3_2_R	77.3 ± 1.8	66.0 ± 0.9	4.3 ± 0.6
M1_3_L	36.4 ± 0.9	10.0 ± 0.3	0.6 ± 0.04
M2_3_L	62.3 ± 1.6	25.0 ± 0.7	1.1 ± 0.09
M3_3_L	50.8 ± 1.2	15.0 ± 0.4	1.1 ± 0.09
M1_3_R	38.3 ± 0.9	61.0 ± 0.9	4.0 ± 0.5
M2_3_R	44.5 ± 1.1	77.0 ± 1.3	6.6 ± 0.8
M3_3_R	39.0 ± 0.9	38.0 ± 0.7	2.1 ± 0.2

**Table 5:** Volatile compound composition (%) in the fresh shoots and roots of *E. maritimum* in vitro samples.

RI *	Compound	Composition Range (Shoots)	Composition Range (Roots)
904	1-Propen-2-ol acetate	n.d.–1.45	1.94–10.27
979	Pentanal	n.d.–0.48	n.d.
1028	α-Pinene	3.42–42.34	n.d.–9.91
1083	Hexanal	n.d.–0.52	n.d.
1117	β-Thujene	0.95–5.19	n.d.
1161	β-Myrcene	4.97–17.76	n.d.–1.19
1167	α -Phellandrene	n.d.–0.10	n.d.–0.98
1202	1-Pentanol	n.d.–0.85	n.d.
1207	Limonene	2.1–7.87	n.d.
1211	β-Phellandrene	n.d.–0.38	n.d.
1246	γ-Terpinene	n.d.–0.43	n.d.
1249	o-Cymene	n.d.–0.47	n.d.
1251	Mesitylene	3.82–15.43	4.1–11.6
1256	3,4-Dimethylstyrene	n.d.–0.84	n.d.
1340	Hemimellitene	0.41–2.81	0.82–2.71
1406	1,3,5,5-Tetramethyl-1,3-cyclohexadiene	0.34–0.89	n.d.–1.01
1462	trans-Limonene oxide	n.d.–0.17	n.d.
1582	Methyl citronellate	0.05–0.31	n.d.
1616	Safranal	0.31–2.29	n.d.–0.78
1640	Benzeneacetaldehyde	n.d.–0.37	n.d.
1663	cis-Verbanol	0.15–0.75	n.d.
1685	p-Mentha-1,5-dien-8-ol	n.d.–0.21	n.d.
1687	trans-Verbenol	0.56–3.16	n.d.
1689	γ-Selinene	0.25–0.51	n.d.
1695	Cadinene	2.68–5.91	3.98–15.58
1710	Germacrene D	n.d.–3.28	n.d.
1729	Valencene I	9.47–19.85	2.25–21.67
1732	Valencene II	n.d.–0.77	n.d.–0.78
1792	Selina-3,7(11)-diene	n.d.–0.16	n.d.–0.51
1822	2-Methyl-2-buteonic acid	n.d.–7.20	n.d.–2.12
1875	Thymoquinone	n.d.–0.80	n.d.–1.19
1896	Duraldehyde	1.38–8.99	11.16–29.61
1929	Mesitaldehyde	6.74–29.17	33.77–48.67
	Sum of unidentified	0.89–10.45	0–27.15

* Retention indexes (RI) determined on the CP-Wax 52CB capillary column., based on NIST (National Institute of Standards and Technology) MS Search 2.2 library. n.d—not detected.

**Table 6:** Plant materials from *E. maritimum* used for phytochemical analyses.

Sample Code	Plant Part	Growth Conditions	Population	Sample Type
EM1 W_L	Leaves	Wild	EM1	Pooled sample of 20 individuals
EM2 W_L	Leaves		EM2	
EM1 F_L	Leaves	Field-grown, first vegetation season	EM1	Pooled sample of 20 individuals
EM2 F_L	Leaves		EM2	
EM1 F_R	Roots		EM1	
EM2 F_R	Roots		EM2	
M1_1_L	Shoot	In vitro	EM2	Sample of individual mericlones
M2_1_L	Shoot			
M3_1_L	Shoot			
M1_1_R	Roots			
M2_1_R	Roots			
M3_1_R	Roots			
M1_2_L	Leaves	Field-grown, first vegetation season	EM2	Sample of individual mericlones
M2_2_L	Leaves			
M3_2_L	Leaves			
M1_2_R	Roots			
M2_2_R	Roots			
M3_2_R	Roots			
M1_3_L	Leaves	Field-grown, second vegetation season	EM2	Sample of individual mericlones
M2_3_L	Leaves			
M3_3_L	Leaves			
M1_3_R	Roots			
M2_3_R	Roots			
M3_3_R	Roots			

**Table 7:** Standard solutions used for quantitative analyses.

Compound	Class	Purity	Mass, mg	Volume, mL	Stock Solution, mg/mL	Calibration Range
Leucine	Amino acids	99%	4.5	10 mL water	0.45	0.1–10 μg/mL R^2^ = 0.998
Chlorogenic acid	Hydroxycinnamic acids	98%	10.1	10 mL methanol	1.01	0.1–100 μg/mL R^2^ = 0.998
Rosmarinic acid	Hydroxycinnamic acids	98%	9.8	10 mL methanol	0.98	0.1–100 µg/mL R^2^ = 0.993

**Table 8:** A summary of the procedures for phytochemical screening using 96-well plate assays.

Carried Test	Procedure	Wavelength	Standard and Concentration Range
Total phenolic content (TPC)	25 µL of extract was mixed with 75 µL of H_2_O and 25 µL of Folin–Ciocalteu reagent (1:10) for 6 min. 100 µL of a 7% Na_2_CO_3_ solution was added; the plate was shaken for 30 s and left in a dark place at room temperature for 90 min.	765 nm	0.025–0.20 mg mL^−1^ gallic acid solutions
Total saponin content (TSC)	20 µL of the extract was mixed with 20 µL of 0.5% anisaldehyde (diluted with ethyl acetate) and allowed to react for 10 min in a dark place. Afterwards, 200 µL of 72% sulfuric acid was added and allowed to react for color development at 60 °C for 10 min.	560 nm	0.10–0.40 mg mL^−1^ escin solutions
Total sugar content (Sugars)	50 µL of extract was mixed with 150 µL of H_2_SO_4_ and 30 µL of 5% phenol reagent before being heated in an oven at 90 °C for 5 min. After heating, the plates were cooled.	490 nm	0.045–0.90 mg mL^−1^ glucose solutions
Antiradical activity/ DPPH radical scavenging (ARA/DPPH)	20 µL of extract was mixed with 180 µL of 150 µM DPPH reagent. The plate was kept in the dark at room temperature for 60 min. Different concentrations of the extract were tested to find the IC50, which is the concentration at which the absorbance of DPPH dropped by 50%.	517 nm	0.018–0.22 μg mL^−1^ ascorbic acid solutions

## Data Availability

The data presented in this study are available on request from the corresponding authors.
